# Motion Segmentation Based on Model Selection in Permutation Space for RGB Sensors

**DOI:** 10.3390/s19132936

**Published:** 2019-07-03

**Authors:** Xi Zhao, Qianqing Qin, Bin Luo

**Affiliations:** The State Key Laboratory of Information Engineering in Surveying, Wuhan University, Wuhan 430079, China

**Keywords:** motion segmentation, subspace clustering, multi-model fitting, permutation preferences

## Abstract

Motion segmentation is aimed at segmenting the feature point trajectories belonging to independently moving objects. Using the affine camera model, the motion segmentation problem can be viewed as a subspace clustering problem—clustering the data points drawn from a union of low-dimensional subspaces. In this paper, we propose a solution for motion segmentation that uses a multi-model fitting technique. We propose a data grouping method and a model selection strategy for obtaining more distinguishable data point permutation preferences, which significantly improves the clustering. We perform extensive testing on the Hopkins 155 dataset, and two real-world datasets. The experimental results illustrate that the proposed method can deal with incomplete trajectories and the perspective effect, comparing favorably with the current state of the art.

## 1. Introduction

Motion segmentation is aimed at segmenting objects with different motions in the video and has become an essential issue for many computer vision applications, such as a visual odometer and video segmentation. A review of motion segmentation can be found in Zappella et al. [1].

In this paper, we propose a robust solution that addresses the issue of motion segmentation. In the case of affine cameras, the trajectories of a rigidly moving object lie in a linear subspace of at most four dimensions, and the trajectories of different objects lie in different subspaces [2,3]. Thus, motion segmentation is equivalent to the clustering of the data into subspaces.

Based on subspace clustering, motion segmentation algorithms were classified into four categories [4,5], i.e., algebraic methods [6,7,8], statistical methods [9,10,11,12], iterative methods [13,14,15], and spectral clustering methods [16,17,18,19,20,21,22,23]. The first three categories’ methods require the dimension and number of subspaces as prior information and are sensitive to the initial values and noise. The spectral clustering methods are effective at data clustering but cannot handle outliers and noise and often require post-processing. In recent years, there are some motion segmentation algorithms based on deep learning [24,25,26,27], which usually obtain more accurate segmentation results. However, the results of the deep-learning-based method depend strongly on the semantic segmentation. Therefore, deep learning methods require a sufficient number of samples and may fail without concrete semantic information. This is different from the motion segmentation problem we consider. There exist some objects without semantic information in actual data. Our proposed solution for motion segmentation does not depend on semantic information. 

Recently, many multi-model fitting methods have been developed to solve the problem of motion segmentation [28,29,30,31,32,33,34]. The multi-model fitting methods first generate a model hypotheses by sampling, and then estimate the model parameters by analyzing the preferences from the point-to-model hypotheses. J-linkage [28] involves constructing preference sets of the points in the conceptual space through the selected inlier thresholds, and then computing the Jaccard distance between each point for bottom-up hierarchical clustering. Kernel fitting (KF) [29] does not directly use residual sequences to represent the data points but instead uses non-descending sorted residual sequences to represent the data points. T-linkage [30,31,32] is an extension of J-linkage, which expands the binary conceptual space into a continuous conceptual space and replaces the Jaccard distance with a Tanimoto distance. The random cluster model simulated annealing (RCMSA) method [33] expresses the point preferences by constructing a weighted graph, and the multi-model fitting task is transformed into a graph cut problem, which can be effectively solved in the simulated annealing framework. Robust multiple model fitting with preference analysis and low-rank approximation (RPA) [34] uses a kernel matrix instead of the Tanimoto metric and combines preference analysis with low-rank approximation, which transforms the multi-structure model fitting problem into multiple single-structure model fitting problems. RPA then uses m-estimator sample consensus (MSAC) to solve the single-structure fitting problem.

Multi-model fitting methods have proven to be robust to noise and outliers. In motion segmentation, there are always multiple motion subspaces, so multi-model fitting methods can solve the motion segmentation problem well and have performed better than other advanced methods [30,31]. In this paper, we also propose a motion segmentation method based on the multi-model fitting technique. We over-segment the data first, then perform model selection and clustering. 

Model selection is largely dependent on the quality of the initial values. However, some over-segmentation methods, such as sequential RANSAC and spectral clustering, are not satisfactory. The former is time-consuming and often leads to inaccurate estimation due to the “fitting-and-removing” framework. The latter is less stable due to the sensitivity to noise. We select locality-sensitive hashing (LSH) for obtaining a series of clusters as the initial model set quickly. By constructing a similarity matrix instead of the original data in performing LSH, we apply the dual preference constraint to data points for accurate assignment. Then, we perform a model selection process to obtain a few high-quality models and calculate the data point residuals to update the similarity between data points. Some methods [29,35,36,37,38,39,40] balance the goodness of fit and the complexity of the model without combining the potential spatial correlation in the data. However, points at relatively close spatial distances should usually be assigned the same label. We adopt energy minimization to introduce the spatial smoothness of the data points and thereby optimize the preference of data points. Meanwhile, we combine with geometric robust information criterion (GRIC) [41] to improve the convergence speed and accuracy of the model selection. In addition, we detect the number of motion models by merging the possible model pairs in model selection. The authors [6,42,43,44,45] put in the number of motion models as prior information, while [46,47] used a complexity or rank measurement to estimate this number, which might lead to wrong estimates about the number of motion models in the presence of noise and outliers. The method in [48] is able to estimate the number of motions automatically, which first over-segments motions by the spectral clustering, then merges the over-segmented motions. However, it has a high computational cost due to the use of a more complex geometric model in a mixed norm optimization scheme. Moreover, the spectral clustering is sensitive to noise. Our method is robust in the presence of outliers, since both energy minimization and GRIC impose penalties on outliers.

The main contributions of this paper are three-fold:

1. We propose a data grouping method, which defines the similarity between data points, and introduce the LSH tool in the processing of the similarity to group the data points;
2. We propose a model selection approach that combines energy minimization and the geometric robust information criterion (GRIC) to optimize the model set obtained by the data grouping;
3. No prior knowledge is needed, such as the number of motions, as this can be automatically estimated through the model selection.

The structure of this paper is as follows. In Section 2 and Section 3, we describe the proposed motion segmentation algorithm in detail. The data grouping process is presented in Section 2, and Section 3 introduces the model selection approach. The experimental results are presented in Section 4. Finally, we draw conclusions in Section 5. 

## 2. Data Grouping in Permutation Space

Before we describe our method, we first briefly review the basic formulation setup in motion segmentation.

Under the affine projection model, it is assumed that an *f*-frame image sequence is extracted from the video. The image sequence is then preprocessed by a feature point extraction algorithm, such as scale-invariant feature transform (SIFT) or speeded-up robust features (SURF), to obtain N tracked feature points ${\left\{\left(x_{\hat{f}n},y_{\hat{f}n}\right)\right\}}_{\hat{f}=1\ldots F}^{n=1\ldots N}$. The 3D coordinates ${\left\{\left(X_{n},Y_{n},Z_{n}\right)\right\}}_{n=1}^{N}$ of the tracking points can then be converted into a 2D representation by Equation (1) [1]:

(1) $$\left[\begin{array}{c} x_{\hat{f}n}\\ y_{\hat{f}n} \end{array}\right]=A_{\hat{f}}\left[\begin{array}{c} X_{n}\\ Y_{n}\\ Z_{n}\\ 1 \end{array}\right],$$

where $A_{\hat{f}}=\left[R_{2\hat{f}\times 3}|T_{2\hat{f}\times 1}\right]\in {\mathbb{R}}^{2\times 4}$ is the affine motion matrix of the $\hat{f}$- frame image sequence. The input of the motion segmentation problem under the affine projection model is a trajectory matrix composed of the 2D coordinates of the N tracked feature points:

(2) $${\left[\begin{array}{llll} x_{11} & x_{12} & \cdots & x_{1N}\\ y_{11} & y_{12} & \cdots & y_{1N}\\ \vdots & \vdots & \ddots & \vdots \\ x_{F1} & x_{F2} & \cdots & x_{FN}\\ y_{F1} & y_{F2} & \cdots & y_{FN} \end{array}\right]}_{2F\times N}={\left[\begin{array}{c} A_{1}\\ \vdots \\ A_{F} \end{array}\right]}_{2F\times 4}{\left[\begin{array}{l} \begin{array}{ccc} X_{1} & \cdots & X_{N} \end{array}\\ \begin{array}{ccc} Y_{1} & \;\cdots & Y_{N} \end{array}\\ \begin{array}{ccc} Z_{1} & \;\cdots & Z_{N} \end{array}\\ \begin{array}{ccc} \;1 & \;\cdots & \;1 \end{array} \end{array}\right]}_{4\times N}.$$

We can write the above equation in the form of $W_{2F\times N}=A_{2F\times 4}S_{4\times N}$, where W is the trajectory matrix [49]. Clearly, $rank(W)=rank(A_{2F\times 4}S_{N\times 4}^{})\leq 4$. That is, in the affine projection model, the N trajectories from m rigid motions all lie in a union of m linear subspaces of dimensions at most four in ${\mathbb{R}}^{2F}$, and similar trajectories from a single rigid motion also lie in the same subspace. Therefore, the motion segmentation problem can be solved by the clustering of the data into subspaces.

### 2.1. Preference Analysis

As stated in [50], the probability of two points having arisen from the same model can be estimated from the residual sorting information. Therefore, given the data point set $X={\left\{x_{i}\right\}}_{i=1}^{N}$, the proposed method starts by shifting the data points to the permutation space. More specifically, firstly, in the manner of random sampling, a large number of hypotheses ${\left\{\theta_{j}\right\}}_{j=1}^{M}$ are generated from X. The residuals for the data points are then computed and stored in the N×M matrix:

(3) $$R=\left[\begin{array}{ccc} r_{1}^{\left(1\right)} & \cdots & r_{1}^{\left(M\right)}\\ \vdots & \ddots & \vdots \\ r_{N}^{\left(1\right)} & \cdots & r_{N}^{\left(M\right)} \end{array}\right],$$

where the rows represent N points and the columns represent M hypotheses. Therefore, for data point $x_{i}$ its absolute residual to all the M hypotheses is the vector $r_{i}$:

(4) $$r_{i}=[r_{i}^{\left(1\right)}\;r_{i}^{\left(2\right)}\;\cdots \;r_{i}^{\left(M\right)}].$$

The preference of $x_{i}$ is then the permutation:

(5) $$\tau_{i}=[\tau_{i}^{\left(1\right)}\;\tau_{i}^{\left(2\right)}\;\cdots \;\tau_{i}^{\left(M\right)}],$$

which sorts $r_{i}$ in ascending order, i.e., $r_{i}^{\left(\tau_{i}^{\left(1\right)}\right)}\leq r_{i}^{\left(\tau_{i}^{\left(2\right)}\right)}\leq \cdots \leq r_{i}^{\left(\tau_{i}^{\left(M\right)}\right)}$. The “coincidence rate” between two preferences $\tau_{i}$ and $\tau_{j}$ is obtained as

(6) $$f(\tau_{i},\tau_{j})=\frac{1}{k}\left|\tau_{i}^{\left(1:k\right)}\cap \tau_{j}^{\left(1:k\right)}\right|,$$

where $\left|\tau_{i}^{\left(1:k\right)}\cap \tau_{j}^{\left(1:k\right)}\right|$ represents the number of identical elements in sets $\tau_{i}^{\left(1:k\right)}$ and $\tau_{j}^{\left(1:k\right)}$. Generally speaking, we are interested in the top *k* permutation preferences rather than a full ranking of the permutation preferences to analyze the data in the model fitting problems, and we set k=M/10. If the coincidence rate is larger, it indicates that data points $x_{i}$ and $x_{j}$ are more similar.

In order to better express the feature that points sharing the same preference may belong to the same structure, we use a positive semi-definite kernel matrix $S\in {\left[0,1\right]}^{N\times N}$ to define the similarity between $x_{i}$ and $x_{j}$:

(7) $$S(i,j)=\exp(-\varepsilon {\left(i,j\right)}^{2}/2),$$

where $\varepsilon (i,j)=1-f(\tau_{i},\tau_{j})$ represents the distance between $x_{i}$ and $x_{j}$.

### 2.2. Data Grouping by Locality-Sensitive Hashing (LSH)

As stated in Section 2.1, the similarity matrix $S\in {\left[0,1\right]}^{N\times N}$ is used to measure the degree of similarity between data points. Therefore, we use $S(i,j)=\left[\begin{array}{ccc} {\hat{s}}_{\left(1,1\right)} & \cdots & {\hat{s}}_{\left(1,N\right)}\\ \vdots & \ddots & \vdots \\ {\hat{s}}_{\left(N,1\right)} & \cdots & {\hat{s}}_{\left(N,N\right)} \end{array}\right]$ instead of the original data $X={\left\{x_{i}\right\}}_{i=1}^{N}$ to redefine each point, where the value on the diagonal is 1 and the point $x_{i}$ is expressed as a similarity permutation vector ${\hat{s}}_{i}=[{\hat{s}}_{\left(i,1\right)}\;{\hat{s}}_{\left(i,2\right)}\;\cdots \;{\hat{s}}_{\left(i,N\right)}]$. We define a concept of “dual similarity”, i.e., if the similarity permutation vectors ${\hat{s}}_{i}$ and ${\hat{s}}_{j}$ are similar, the data points $x_{i}$ and $x_{j}$ have a high probability of belonging to the same motion model. That is to say, grouping the data points by similarity is a feasible solution.

Locality-sensitive hashing (LSH) is an approximate nearest neighbor search tool, as stated in [51], and hashes high-dimensional points into buckets based on locality, where points of high similarity are hashed into the same LSH bucket. However, if we directly use LSH to hash the data points by Euclidean distance, as in [51], this will result in the points in an identical bucket most likely belonging to different motion models. We therefore use the similarity matrix instead of the original data, which is equivalent to applying a preference constraint to the data points. This is done so that the points in an identical bucket have a high probability of belonging to the same motion model. We adopt the same *p*-stable based LSH as in [51] to process the Euclidean distance between the similarity permutation vectors, to complete the initial grouping of the original data points.

*P*-stable LSH is a locality-sensitive hashing method based on the *p*-stable thought, which calculates the hash values $h_{1}$ and $h_{2}$ of the eigenvectors $v_{1}$ and $v_{2}$, where $v_{1}$ and $v_{2}$ are the eigenvectors of the similarity permutation vectors ${\hat{s}}_{i}$ and ${\hat{s}}_{j}$, respectively. Since the hash function is locally sensitive, if the two eigenvectors $v_{1}$ and $v_{2}$ are closer together, the probability that the hash values $h_{1}$ and $h_{2}$ map to the same bucket will be larger, and vice versa.

The hash function *p*-stable LSH is defined as follows:

(8) $$h_{a,b}(x)=\left\lfloor \frac{a\cdot x+b}{w}\right\rfloor ,$$

where ⌊⋅⌋ is the round down function, each entry in vector a is chosen independently from a *p*-stable distribution, w is a constant greater than 0, and b is a real number chosen uniformly from the range [0,w]. For a detailed description of *p*-stable LSH, see [52].

**Figure 1:**
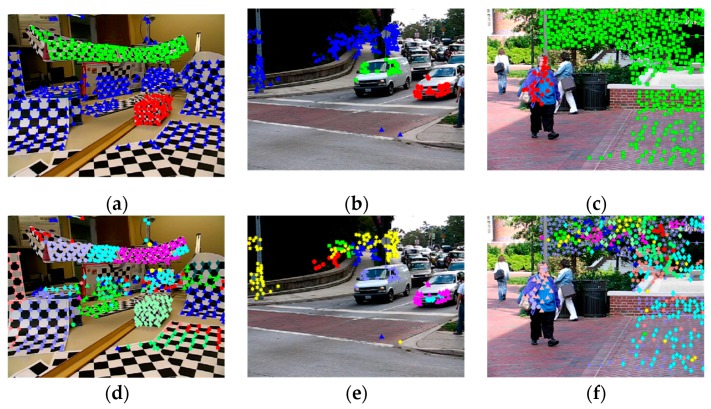
Some data grouping results in video sequences of the Hopkins 155 dataset. Top (**a**–**c**): Ground truth for the checkerboard sequence 2RT3RCT_B, the traffic sequence cars9, and the articulated sequence people2, respectively, where the data points belonging to different motion models are labeled with different colors. Bottom (**d**–**f**): The corresponding data point grouping results. We obtain many data clusters and points in the same cluster almost always belong to the same motion mode.

In order to prevent the existence of small clusters (data points less than the minimal sample sets (MSS)), we first choose a small number of high-density buckets as [51], which contain a significant portion of the data. Because points with high similarity have a high probability of being assigned to the same bucket, these buckets can be used to represent the initial model clusters $C=\left\{c_{1},c_{2},\cdots ,c_{t}\right\}$. These models are then spread to the rest of the data points in a top-down fashion, i.e., we map each data point to its closest model. Finally, we obtain data clusters containing all the data points, as shown in Figure 1, where points in an identical cluster belong to the same motion model. Therefore, this approach can provide a good initialization for the iterative process in the model selection.

## 3. Model Selection

The number of models in the initial model set $C=\left\{c_{1},c_{2},\cdots ,c_{t}\right\}$ obtained by LSH is redundant, so we use a strategy combining energy minimization and the GRIC criterion to select the model that best fits the data.

Firstly, with random sampling in $c_{i}$, the MSS contains almost no outliers, and the generated hypothesis is more likely to be a good fit to the data. We then use energy minimization to select the hypothesis that best fits the cluster. 

We adopt the energy E composed of the data energy $E_{d}$ and smoothness energy $E_{s}$ to measure the quality of the fitting:

(9) $$E=E_{d}+E_{s}.$$

The data term $E_{d}$ is used to penalize inaccuracies induced by the point-to-model assignment, and is generally defined as

(10) $$E_{d}=\sum_{i=1}^{N}D(x_{i},f_{i}),$$

where D is a distance function between point $x_{i}$ and the model hypothesis. 

If we let N denote the set of all such neighboring data point pairs, the smoothness energy is:

(11) $$E_{s}=\sum_{<i,j>\in N}V(f_{i},f_{j}).$$

$V(f_{i},f_{j})$ is derived from the Potts model:

(12) $$V(f_{i},f_{j})=\{\begin{array}{cc} 0 & if\;f_{i}=f_{j}\\ 1 & if\;f_{i}\neq f_{j} \end{array},$$

which penalizes $f_{i}\neq f_{j}$ of the points in a neighborhood.

The minimization of Equation (9) can be optimized effectively with the α-expansion algorithm [53].

After the initial selection by energy minimization, we obtain t redundant models and then select n (n≤t) models that best explain the input data using GRIC. GRIC is a model selection algorithm that establishes a scoring mechanism to rate each model, allowing us to select the model with the lowest score. The GRIC criterion can robustly select the motion model and detect the presence of outliers and is defined as follows:

(13) $$GRIC=\sum_{i}\rho (e_{i}^{2})+(\lambda_{1}dn+\lambda_{2}k).$$

The first term is the error function, which is defined according to the Huber function [54] as

(14) $$\rho (e_{i}^{2})=\{\begin{array}{cc} \frac{e^{2}}{\sigma^{2}} & \frac{e^{2}}{\sigma^{2}}<2.0(r-d)\\ 2.0(r-d) & \frac{e^{2}}{\sigma^{2}}\geq 2.0(r-d) \end{array},$$

where $e_{i}$ represents the residual of the point, and (r−d) is a codimension of the *r*-dimensional points fitted by a manifold of dimension *d*. It can be seen that the error function represents the goodness of fit.

The term $\left(\lambda_{1}dn+\lambda_{2}k\right)$ in Equation (10) represents a penalty on the complexity of the model. $\lambda_{1}dn$ is a penalty term for the dimensionality of the model, where the greater the dimension of the model, the greater the penalty. $\lambda_{2}k$ is a penalty term for the number of parameters of the model, to greater penalize models with more parameters [41]. Therefore, the model GRIC selects is the one with the highest information content, but the least complexity. In addition, we set the penalty factors $\lambda_{1}$ and $\lambda_{2}$ as $\lambda_{1}=\log(4)=1.4$ and $\lambda_{2}=\log N=\log4n$, where n is the number of data points, and k is the number of parameters of the fitted model.

**Figure 2:**
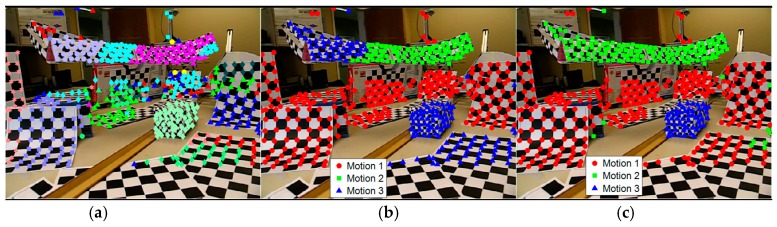
Model selection results obtained on checkerboard sequence 2RT3RCT_B. (**a**) Data grouping results obtained by Equation (7), which is the initial input of the model selection; (**b**) intermediate results of model selection during iterations; (**c**) final segmentation results obtained after model selection.

The energy minimization and GRIC are conducted alternately and continuously until the model set is almost unchanged. Figure 2 shows the model selection results on the 2RT3RCT_B sequence by the proposed model selection approach. As can be seen from Figure 2c, the selected models are very similar to the real model. 

## 4. Model Clustering

Through the model selection, we obtain the number of models and the data point permutation preference information represented by the residual matrix R. The similarity matrix S of the data points is derived from the residual matrix R according to the steps in Section 2.1, which can express the data point permutation preferences well. Since permutation preferences for the points have been proven to be able to distinguish inliers belonging to different models (“model” refers to subspace in motion segmentation) [55,56], bottom-up linkage clustering is adopted in the permutation space for clustering the points. Therefore, points with similar permutation preferences can be sampled to generate good hypotheses, and good hypotheses can make the permutation preferences more distinguishable, thereby improving the clustering.

We present the detailed steps in Algorithm 1.

**Algorithm 1:** Motion Segmentation Algorithm **Input:**  X
*// dataset***Output:**
M
*// clusters of point belonging to the same model*1: S = PermutationSpace (X) *// get the similarity matrix*2: $C=\left\{c_{1},c_{2},\cdots ,c_{t}\right\}$ = LSH (S) *// get the initial model set*3: **Repeat**4:   $\theta_{i}$ = RandomSampling ($c_{i}$)5:   $\Theta =\{\{\theta_{1}^{j}{\}}_{1}^{10},\ldots ,\{\theta_{t}^{j}{\}}_{1}^{10}\}$ = AscendSort (${\left\{\theta_{i}\right\}}_{1}^{t}$) *// sort*
$\theta_{i}$
*by ascending order according to the residuals and extract the top-10 hypotheses*6:   $C^{†}=\{c_{1}^{†},\ldots ,c_{t}^{†}\}$ = α-expansion (Θ) *// select the best-quality hypothesis in each cluster*7:   $C^{*}=\{c_{1}^{*},\ldots ,c_{n}^{*}\}$ = GRIC ($C^{†}$) *//*
*select the model fitting the data best,*
*where*
n≤t
8:        **Until**
$C^{*}$ is not changed. $C^{*}:=C$
9:        
$M=\{M_{1},\ldots ,M_{n}\}$ = LinkageClustering (n,R) *// n is the estimated number of motions, R is the residual information of the data points*

## 5. Experiments

To test the performance of the proposed method, we carried out motion segmentation experiments on the Hopkins 155 dataset [57] and two real-world datasets. We evaluated the performance in terms of the classification error [57].

### 5.1. Results of the Hopkins 155 Dataset

The Hopkins 155 dataset contain 155 video sequences, where 120 of the videos have two motions and 35 of the videos have three motions. In addition, it contains complex motion scenes, with many noise points and isolated points. The sequences can be roughly divided into three categories: Checkerboard sequences, traffic sequences, and articulated sequences. 

We compare the proposed method with the state-of-the-art approaches of random sample consensus (RANSAC) [9], generalized principal component analysis (GPCA) [6], local subspace affinity (LSA) [17], agglomerative lossy compression (ALC) [12], the sparse subspace clustering algorithm (SSC) [5], J-linkage [28], and T-linkage [30]. The average and median classification errors of the different scenes are listed in Table 1 and Table 2, and the average and median classification errors of the other methods are obtained from [19,30]. Note that in order to obtain satisfactory results, our method only requires to tune one parameter (permutation length), which is much fewer than many other state-of-art methods.

**Figure 3:**
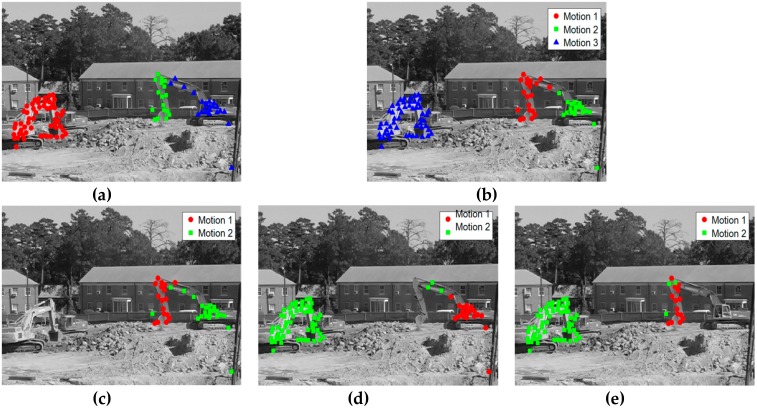
The results of motion segmentation on the two_cranes video sequence. (**a**) Ground truth of the two_cranes sequence, where the red dot represents the first motion model, the green dot represents the second motion model, and the blue dot represents the third motion model; (**b**) the segmentation result with three motions; (**c**) the segmentation result with two motions, which includes the second motion model and the third motion model; (**d**) the segmentation result with two motions, which includes the first motion model and the third motion model; (**e**) the segmentation result with two motions, which includes the first motion model and the second motion model.

We can make the following observations from the two tables. The RANSAC, GPCA, LSA, and ALC methods have high classification error in the entire experiment. Meanwhile, the SSC method always performs well—even on the challenging sequence articulated, the classification error is only 1.42% for three motions and 0.62% for two motions. However, the proposed method performs the best among all the methods on the checkerboard and traffic sequences, obtaining the lowest classification error. The classification error has been significantly reduced, about 12 times better than the best result previously reported by SSC. On the articulated sequences, it scores second-best, and is fairly close to the SSC algorithm. However, the classification error of the proposed method is still much lower than that of the other methods. Moreover, most of the existing methods do not perform well on the articulated sequences. This is because motions in the two_cranes video sequence are very complex and partially dependent on each other (as shown in Figure 3). We can make the observation from Figure 3a that the number of tracking points is only 94, making it impossible to generate sufficient assumptions for good permutation preferences. Figure 3b is the segmentation result of three motions, whose classification error is 3.29%. Figure 3c–e gives the segmentation results of two motions, with a classification error of 5.13%, 3.9%, and 4.17%, respectively.

**Figure 4:**
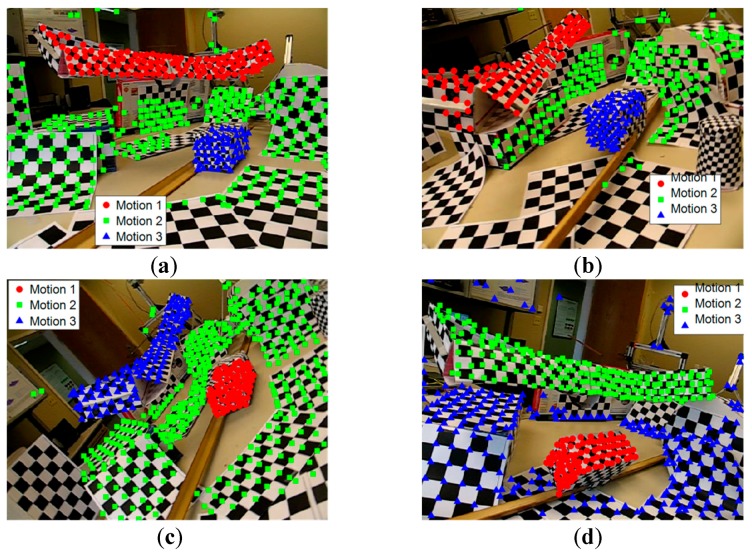

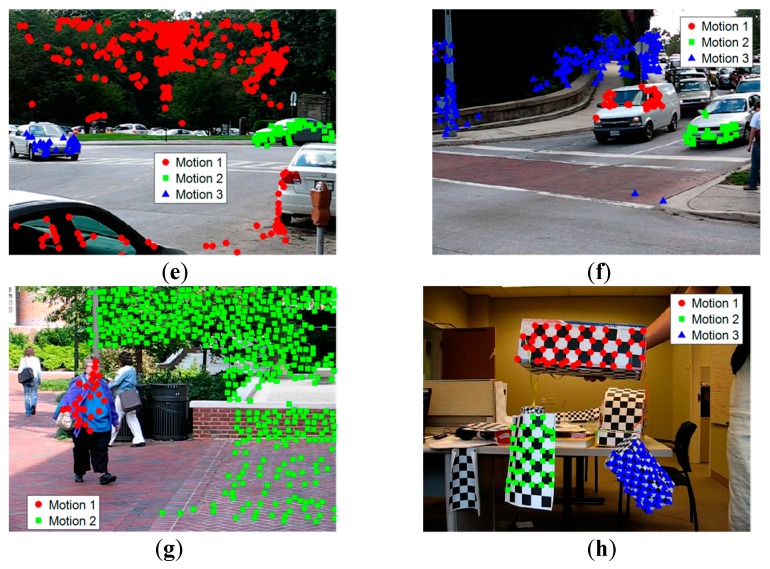
Sample results in video sequences of the Hopkins 155 dataset with the proposed method, with the different motions labeled with points of different colors and shapes. (**a**) 2RT3RCT_B, (**b**) 2RT3RTCRT, (**c**) 2T3RCR, (**d**) 2R3RTC, (**e**) cars5, (**f**) cars9, (**g**) people2, (**h**) articulated.

Figure 4 shows some example frames from the Hopkins 155 dataset, which is the corresponding correct segmentation obtained by our method. The proposed method can correctly classify the points belonging to different motions. Figure 4a–d gives the checkerboard video sequences, Figure 4e,f gives the traffic video sequences, and Figure 4g,h gives the articulated video sequences. It is very difficult for many methods to correctly segment motion models that are close in the spatial domain because they involve the spatial constraints of data points, such as in sampling and clustering. On the contrary, since we group the data points based on similarities in the feature space, instead of grouping the data points with Euclidean distance directly in the Euclidean space, the spatial constraint is not so important for motion model grouping. Therefore, our method can well segment motion models that are spatially close. 

### 5.2. Results of the Real-World Dataset

The Hopkins155 dataset has some limitations, such as limited depth reliefs and dominant camera rotations. Taking into account these limitations, it is not appropriate to use this dataset as a benchmark for investigating motion segmentation capability in the wild [58]. Real-world sequences contain real challenges, such as missing data, unknown number of motions, and perspective effects [48]. For this reason, we also evaluated the proposed method on the real-world datasets: The MTPV62 dataset [48] and the KITTI 3D Motion Segmentation Benchmark (KT3DMoSeg) [58].

The MTPV62 dataset comprises 62 video sequences, of which 50 are from Hopkins 155. Another 12 video sequences have heavy occlusions, of which four video sequences are from [54] and another eight video sequences are provided by [48]. Of the 62 video sequences, 26 contain two motions, 36 contain three motions, 12 suffer from seriously missing data, and nine have strong perspective effects. The KT3DMoSeg dataset is a more challenging dataset because it contains strong perspectives and strong forward translations. All sequences of KT3DMoSeg involve strong perspective effects in the background, but the foreground moving objects often have limited depth reliefs [58]. 

**Figure 5:**
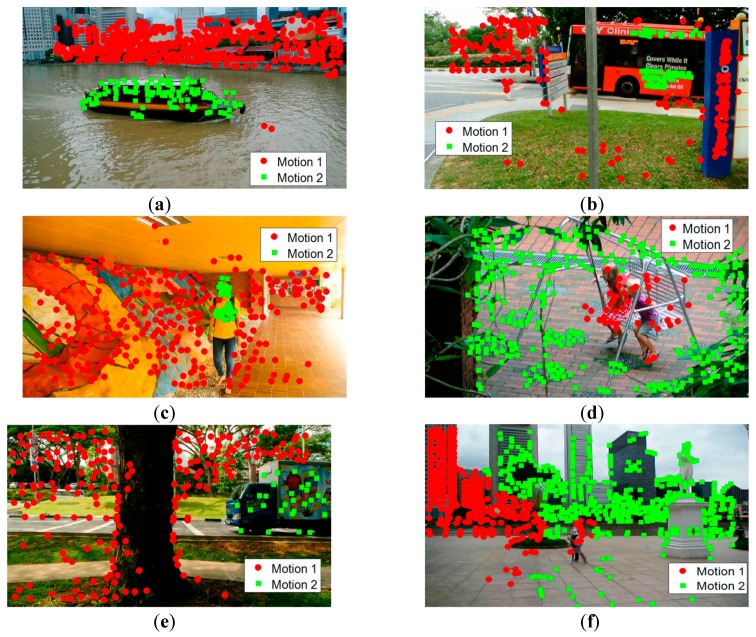
Sample results in video sequences of the MTPV62 dataset with the proposed method, with the different motions labeled with points of different colors and shapes. (**a**) Boat, (**b**) Bus, (**c**) Girl, (**d**) Swing, (**e**) Van, (**f**) Raffles.

**Figure 6:**
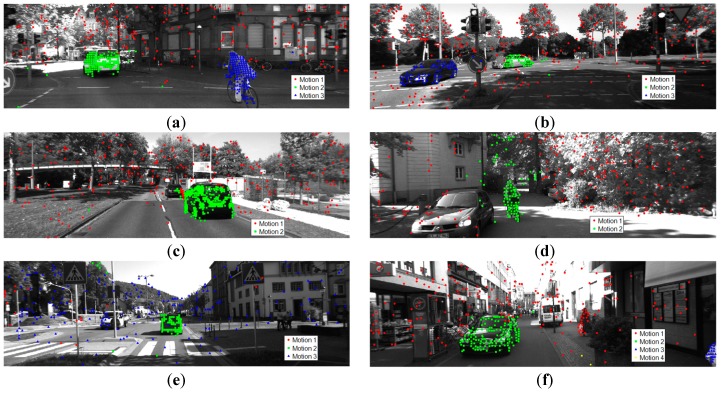
Sample results in video sequences of the KT3DMoSeg dataset with the proposed method, with the different motions labeled with points of different colors and shapes. (**a**) Seq005_Clip01, (**b**) Seq009_Clip03, (**c**) Seq013_Clip02, (**d**) Seq113_Clip01, (**e**) Seq005_Clip01, (**f**) Seq071_Clip01.

We compare the performance of the proposed method with seven state-of-the-art methods: ALC, GPCA, LSA, SSC, TPV [48], LRR [59], and MSSC [60]. The quantitative results are presented in Table 3. All the classification errors of the seven methods were obtained from [58]. We use Chen’s matrix completion approach [61] to handle missing data. Some qualitative results are presented in Figure 5 and Figure 6.

We make the following observations from Table 3. First, we achieved a pretty good performance on Hopkins 50 clips. However, the average classification error on the Missing Data 12 clips is a little high. As seen in Figure 5f, incorrect segmentation on the Raffles sequence results in the high classification error of MTPV62 dataset. Actually, the classification error on the Raffles sequence is as high as 31.33%. The reason is that the distribution of the inliers of the foreground and background is extremely unbalanced, and the background is very complicated. In addition, there are only seven points belonging to the foreground, which results in difficulty in sampling an all-inlier minimal set and seriously impacts the performance of the preferences. Secondly, we obtained the best average classification error on the KT3DMoSeg dataset. However, the segmentation accuracy can be further increased when considering the complexity of KT3DMoSeg. Many background objects in Figure 6 have noncompact shapes, thus the background is often separated and the segmentation on the junction of the foreground and background is very difficult. The most obvious case is Figure 6d. In addition, we adopt a single geometric model in handling the motion segmentation problem. However, the comparison in [58] shows that the performance of multi-view approaches is consistently better than when we adopt a single geometric model. Sometimes subspace overlap occurs with a single geometric model. Just as presented in Figure 6e,f, some foreground objects are incorrectly segmented into the background. 

## 6. Conclusions

In this paper we have proposed a robust subspace clustering method that applies multi-model fitting to the problem of motion segmentation. We first transformed the data into permutation space and then defined a similarity matrix based on data point permutation preferences and used this in grouping and clustering the data points. Then, we used a model selection strategy that combines energy minimization and the GRIC information criterion to select the best model, which can generate more distinguishable permutation preferences for the data points, thereby obtaining better clustering results. In the experiments undertaken in this study, the proposed method can deal with incomplete trajectories and perspective effect, achieving state-of-the-art performance in motion segmentation. 

## Figures and Tables

**Table 1:** Classification errors (%) for sequences with three motions.

Methods	RANSAC	GPCA	LSA 4n	ALC 5	SSC	J-Lnkg	T-Lnkg	Proposed
**Checkerboard: 26 sequences**								
Mean	25.78	31.95	5.80	6.78	2.97	8.55	7.05	0.17
Median	26.00	32.93	1.77	0.92	0.27	4.38	2.46	0.00
**Traffic: 7 sequences**								
Mean	12.83	19.83	25.07	4.01	0.58	0.97	0.48	0.08
Median	11.54	19.55	23.79	1.35	0.00	0.00	0.00	0.00
**Articulated: 2 sequences**								
Mean	21.38	16.85	7.25	7.25	1.42	9.04	7.97	1.65
Median	21.38	16.85	7.25	7.25	0.00	9.04	7.97	1.65
**All: 35 sequences**								
Mean	22.94	28.66	9.73	6.26	2.45	7.06	5.78	0.24
Median	22.03	28.26	2.33	1.02	0.20	0.73	0.58	0.00

**Table 2:** Classification errors (%) for sequences with two motions.

Methods	RANSAC	GPCA	LSA 4n	ALC 5	SSC	J-Lnkg	T-Lnkg	Proposed
**Checkerboard: 78 sequences**								
Mean	6.52	6.09	2.57	2.56	1.12	1.20	7.05	0.02
Median	1.75	1.03	0.27	0.00	0.00	0.00	2.46	0.00
**Traffic: 31 sequences**								
Mean	2.55	1.41	5.43	2.83	0.02	0.70	0.02	0.00
Median	0.21	0.00	1.48	0.30	0.00	0.00	0.00	0.00
**Articulated: 11 sequences**								
Mean	7.25	2.88	4.10	6.90	0.62	0.82	7.97	0.82
Median	2.64	0.00	0.22	0.89	0.00	0.00	7.97	0.00
**All: 120 sequences**								
Mean	5.56	4.59	3.45	3.03	0.82	1.62	0.86	0.09
Median	1.18	0.38	0.59	0.00	0.00	0.00	0.00	0.00

**Table 3:** Classification errors (%) for sequences with MTPV62 dataset and KT3DMoSeg dataset.

Methods	MTPV62				KT3DMoSeg	
State of the Art	Perspective 9 clips	Missing Data 12 clips	Hopkins 50 clips	All 62 clips	Average	Median
LSA	-	-	-	-	38.30	38.58
GPCA	40.83	28.77	16.20	16.58	34.60	33.95
ALC	0.35	0.43	18.28	14.88	24.31	19.04
SSC	9.68	17.22	2.01	5.17	33.88	33.54
TPV	0.46	0.91	2.78	2.37	-	-
LRR	-	-	-	-	33.67	36.01
MSSC	-	0.65	0.65	0.65	-	-
Proposed	-	3.36	0.16	0.78	23.69	23.97
